# Effects of tumour necrosis factor α upon the metabolism of the endocannabinoid anandamide in prostate cancer cells

**DOI:** 10.1371/journal.pone.0185011

**Published:** 2017-09-14

**Authors:** Jessica Karlsson, Sandra Gouveia-Figueira, Mireille Alhouayek, Christopher J. Fowler

**Affiliations:** 1 Department of Pharmacology and Clinical Neuroscience, Pharmacology Unit, Umeå University, Umeå, Sweden; 2 Department of Chemistry Umeå University, Umeå, Sweden; Faculty of Medicine & Health Science, UNITED ARAB EMIRATES

## Abstract

Tumour necrosis factor α (TNFα) is involved in the pathogenesis of prostate cancer, a disease where disturbances in the endocannabinoid system are seen. In the present study we have investigated whether treatment of DU145 human prostate cancer cells affects anandamide (AEA) catabolic pathways. Additionally, we have investigated whether cyclooxygenase-2 (COX-2) can regulate the uptake of AEA into cells. Levels of AEA synthetic and catabolic enzymes were determined by qPCR. AEA uptake and hydrolysis in DU145 and RAW264.7 macrophage cells were assayed using AEA labeled in the arachidonic and ethanolamine portions of the molecule, respectively. Levels of AEA, related *N*-acylethanolamines (NAEs), prostaglandins (PG) and PG-ethanolamines (PG-EA) in DU145 cells and medium were quantitated by ultra-performance liquid chromatography-tandem mass spectrometry (UPLC–MS/MS) analysis. TNFα treatment of DU145 cells increased mRNA levels of *PTSG2* (gene of COX-2) and decreased the mRNA of the AEA synthetic enzyme *N*-acyl-phosphatidylethanolamine selective phospholipase D. mRNA levels of the AEA hydrolytic enzymes fatty acid amide hydrolase (FAAH) and *N*-acylethanolamine-hydrolyzing acid amidase were not changed. AEA uptake in both DU145 and RAW264.7 cells was inhibited by FAAH inhibition, but not by COX-2 inhibition, even in RAW264.7 cells where the expression of this enzyme had greatly been induced by lipopolysaccharide + interferon γ treatment. AEA and related NAEs were detected in DU145 cells, but PGs and PGE_2_-EA were only detected when the cells had been preincubated with 100 nM AEA. The data demonstrate that in DU145 cells, TNFα treatment changes the relative expression of the enzymes involved in the hydrolytic and oxygenation catabolic pathways for AEA. In RAW264.7 cells, COX-2, in contrast to FAAH, does not regulate the cellular accumulation of AEA. Further studies are necessary to determine the extent to which inflammatory mediators are involved in the abnormal endocannabinoid signalling system in prostate cancer.

## Introduction

The endocannabinoid (eCB) system consists of a group of endogenous signaling molecules, primarily the arachidonic acid derivatives anandamide (arachidonylethanolamide, AEA) and 2-arachidonoyl glycerol (2-AG), two receptors (cannabinoid receptors 1 and 2 [CB_1_ and CB_2_]) and the enzymes responsible for the synthesis and breakdown of these ligands. The eCB system is present throughout the body, and is involved in a variety of regulatory and homeostatic functions including neuromodulation, control of feeding, pain and inflammation, bone turnover, reproduction and cell survival [1].

AEA belongs to a family of *N*-acylethanolamine (NAE) lipids, which also include the endogenous anti-inflammatory agent palmitoylethanolamide (PEA) and the satiety factor oleoylethanolamide (OEA). These lipids are synthesized on demand from phosphatidylethanolamides by multiple routes [2], although an important enzyme in this respect is *N*-acyl-phosphatidylethanolamine selective phospholipase D (NAPE-PLD) [3,4]. Normally, the relative levels of NAEs reflect the corresponding tissue levels of the phosphatidylethanolamide precursor lipids, and in mammalian brains, for example, AEA comprises only ~1% of the total NAE content (expressed in mol%) in contrast to PEA (~30%) and OEA (~12%) [5]. In contrast, in human seminal plasma, AEA levels are much higher (12 nM) relative to PEA and OEA levels (32 and 33 nM) [6].

NAEs are hydrolysed to their corresponding fatty acids by the enzymes fatty acid amide hydrolase (FAAH) and *N*-acylethanolamine-hydrolyzing acid amidase (NAAA). The two enzymes have different cellular localisations, pH optima, substrate specificities and inhibitor sensitivities. Additionally, AEA can be oxidised by cytochrome P450 enzymes, by lipoxygenases and not least by cyclooxygenase-2 (COX-2, *PTGS2*) to produce biologically active products [2,7]. In the case of COX-2, the products are prostaglandin ethanolamides (PG-EAs), which have both pro- and anti-inflammatory properties [7]. The cellular accumulation of AEA is regulated by the FAAH activity of the cells. Thus, transfection of cells with FAAH increases the rate of AEA uptake, while its inhibition reduces the uptake [8]. This reflects the ability of the enzyme to reduce the intracellular AEA concentration and hence maintain its extra-: intracellular gradient as a driving force for uptake. It is not known whether COX-2 can fulfil the same function. This could be of considerable importance in inflamed and tumour tissue, where COX-2 is overexpressed (see [9,10] for examples for prostate cancer).

The eCB system is implicated in cancer and tumor progression. Thus, eCBs have been shown to affect proliferation, invasive properties, migration and adhesion of human cancer cells, including prostate cancer cells [11–14]. Human androgen-sensitive LNCaP cells express FAAH, and it knockdown with siRNA reduces the invasivity of these cells across Matrigel-coated transwells in response to fibroblast conditioned media [15]. Conversely, FAAH transfection of human androgen-insensitive PC-3 prostate cancer cells, which normally express low levels of this enzyme, increases their invasivity in the same system [15].

Evidence is accruing to show that AEA metabolism is disturbed in prostate cancer, and that this can contribute to disease outcome. NAPE-PLD is expressed by prostate cancer cells [16], and in a small (N = 4) set of prostate tumour biopsies, AEA levels ranged from 13 to 48 mol% of the total NAEs [17]. For comparison, AEA levels are ~2 mol% of the total NAEs in human glioblastoma samples [18]. In a large set of tumour samples obtained at diagnosis, a high FAAH expression was found to be associated with the severity of the disease [19]. NAAA, which is expressed in the prostate at higher levels than in other tissues [15], is also associated with aggressiveness of prostate tumours [20]. Taken together, these data suggest that AEA turnover is abnormal in prostate tumour cells. However, it is not clear why this is the case.

Inflammation plays a major role in the pathogenesis of cancer [21], raising the possibility that the disturbances in AEA turnover are secondary to aberrant regulation of the synthetic and metabolic enzymes by inflammatory components. One potential candidate for this is tumour necrosis factor α (TNFα). TNFα, despite its name, has many pro-tumour actions [22], and high tumour levels of this cytokine are associated with a poor prognosis in prostate cancer [23]. Knockout of TNFα type 1 receptor reduces the incidence of prostate tumours induced by repeated *N*-methyl *N*-nitrosurea + testosterone treatment in mice [24]. Although there is good evidence that AEA can affect TNFα levels in immune cells (see e.g. [25,26]), little is known as to whether TNFα can affect AEA turnover. However, intracerebroventricular injection of TNFα increases [^14^C]AEA synthesis from [^14^C]arachidonic acid in medialbasal hypothalamus homogenates [27]. Conversely, plasma AEA levels in cholestatic bile duct ligated mice are reduced by anti-TNFα treatment or by genetic deletion of TNFα [28]. Nothing is known as to whether TNFα affects the eCB system in prostate cancer cells. In consequence, we have investigated the effects of TNFα treatment upon the enzymes involved in AEA synthesis and metabolism in human androgen-independent DU145 prostate cancer cells. These cells were chosen because they are responsive to TNFα [29], not least showing an increased expression of COX-2 after treatment with this cytokine [30]. An additional aim of the project has been to determine whether a changed expression of COX-2 affects the cellular accumulation of AEA.

## Materials and methods

### Materials

[Ethanolamide-1-^3^H]AEA ([Et-^3^H]AEA), [arachidonyl-5,6,8,9,11,12,14,15-^3^H]AEA ([Ara-^3^H]AEA), [15-^3^H]17-phenyl trinor prostaglandin F_2α_ ethyl amide ([^3^H]bimatoprost) and [5,6,8,9 11,12,14,15-^3^H(*N*)]arachidonic acid ([^3^H]AA) were obtained from American Radiolabeled Chemicals, Inc (St Louis, MO, USA). [5,6,8,9,12,14,15-^3^H]prostaglandin F_2α_ ([^3^H]PGF_2α_) was obtained from Perkin Elmer (Waltham, USA). Anandamide, arachidonic acid, bimatoprost, PGF_2α_ and URB597 (cyclohexylcarbamic acid 3′-carbamoylbiphenyl-3-yl ester) were purchased from Cayman Chemical Co. (Ann Arbor, MI, USA). Recombinant human TNFα was obtained from R&D systems (Abingdon, UK). Recombinant mouse interferon-γ (INFγ) was obtained from Merck Millipore (Darmstadt, Germany). γ-Irradiated lipopolysaccharide (LPS) from *E*. *coli* serotype O111:B4 was obtained from Sigma Aldrich (St Louis, MO, USA). Penicillin and streptomycin were bought from ThermoFisher Scientific (Waltham, USA). For the qPCR experiments, primers (Table 1) were bought from Integrated DNA Technologies (Leuven, Belgium). For the targeted lipidomic experiments, and for the quantified values presented here, the following native and deuterated standards were purchased from Cayman Chemicals: AEA, PEA, OEA, LEA, 2-AG, thromboxane B_2_ (TXB_2_), PGE_2_-ethanolamide (PGE_2_-EA), PGE_2_-EA-d_4_, AEA-d_8_, PEA-d_4_, OEA-d_4_, PGF_2α_, PGE_2_, PGD_2_, 2-AG-d_8_, 15-hydroxyeicosatetraenoic acid (15-HETE), 20-HETE, PGE_2_-d_4_, PGD_2_-d_4_, 5-HETE-d_8_, 20-HETE-d_6_ (internal standard for 15-HETE), TXB_2_-d_4_, 12-[[(cyclohexylamino)carbonyl]amino]-dodecanoic acid (CUDA) and butylhydroxytoluene (BHT)). Protease inhibitor cocktail III was purchased from Merck Chemicals and Life Science AB (Solna, Sweden). All solvents and chemicals were of HPLC grade or higher. Water was purified by a Milli-Q Gradient system (Millipore, Milford, MA, USA).

**Table 1:** Primer sequences used in the present study.

Species	Gene	Product	Primer sequence
	*RPL19*	RPL19	Fwd: CACATCCACAAGCTGAAGGCA
			Rev: CTTGCGTGCTTCCTTGGTCT
	*NAPEPLD*	NAPE-PLD	Fwd: ACTGGTTATTGCCCTGCTTT
			Rev: AATCCTTACAGCTTCTTCTGGG
	*FAAH*	FAAH	Fwd: CACACGCTGGTTCCCTTCTT
			Rev: GGGTCCACGAAATCACCTTTGA
	*NAAA*	NAAA	Fwd: ATGGAGCGTGGTTCCGAGTT
			Rev: CTGAGGTTTGCTTGTCCT
	*PTGS1*	COX-1	Fwd: GCAGCCCTTCAATGAGTACC
			Rev: TGCCATCTCCTTCTCTCCTAC
	*PTGS2*	COX-2	Fwd: AGCAGGCAGATGAAATACCAG
			Rev: ACCAGAAGGGCAGGATACA
Human	*cPLA2g4a*	PLA_2_	Fwd: CAGCTGTAGCAGATCCTGATG
			Rev: TAAATGTGAGCCCACTGTCC
	*DAGLa*	DAGLα	Fwd: CCCAAATGGCGGATCATCG
			Rev: GGCTGAGAGGGCTATAGTTAGG
	*DAGLb*	DAGLβ	Fwd: TCAGGTGCTACGCCTTCTC
			Rev: TCACACTGAGCCTGGGAATC
	*MGLL*	MAGL	Fwd: GGAAACAGGACCTGAAGACC
			Rev: ACTGTCCGTCTGCATTGAC
	*ABHD6*	ABHD6	Fwd: GATGTCCGCATCCCTCATAAC
			Rev: CCAGCACCTGGTCTTGTTTC
	*ABHD12*	ABHD12	Fwd: CCTGTAGCCAAGGTCTGAATG
			Rev: GGCAGAAAGCTCTATAGCATCG
	*Rpl19*	RPL19	Fwd: TGACCTGGATGAGAAGGATGAG
			Rev: CTGTGATACATATGGCGGTCAATC
	*Napepld*	NAPE-PLD	Fwd: GACCCAGAAGATGCTGTAAGG
			Rev: CTGGCGGCTCTAGGTAATG
	*Faah*	FAAH	Fwd: AGAGTAGGAGTATCAGGGAGTG
Mouse			Rev: CATCAGCAGCGTTTAAGTCG
	*Naaa*	NAAA	Fwd: ATTATGACCATTGGAAGCCTGCA
			Rev: CGCTCATCACTGTAGTATAAATTG
	*Ptgs2*	COX-2	Fwd: AGATTCCCTCCGGTGTTTG
			Rev: CCCTTCTCACTGGCTTATGTAT

### Cell culture

Human DU145 prostate cancer cells were obtained from the American Type Culture Collection (Manassas, VA, USA). They were cultured in Eagle´s minimum essential medium supplemented with non-essential amino acids, 2 mM L-glutamine, 10% foetal bovine serum (FBS) and 100U ml^-1^ penicillin and 100μg ml^-1^ streptomycin and used over a passage range of 15–31. RAW264.7 mouse macrophage cells (European collection of cell cultures, Porton Down, UK) were cultured in Dulbeccos modified Eagle -high glucose medium supplemented with 10% FBS, 100U ml^-1^ penicillin and 100μg ml^-1^ streptomycin and used over a passage range of 14–31.

### TNFα treatment of DU145 cells

DU145 cells were cultured in 75cm^2^ flasks at 37°C with 5% CO_2_ at humidified atmospheric pressure and split (ratio 1:3–4) approximately twice a week. For the PCR, uptake and hydrolysis assays, the cells (1.75 x 10^5^/well) were seeded into 24 well plates and allowed to attach for 4–6 h. The medium was changed to serum-free and the cells were allowed to equilibrate over night. The next day the cells where exposed to either TNFα (20 ng ml^-1^ final concentration) or vehicle (PBS supplemented with 0.001% w/v human serum albumin, final concentration) in serum-free medium and incubated for 0–4 h, as appropriate. This concentration of TNFα has been shown to induce COX-2 in DU145 cells [28]. For the lipidomic study a similar protocol was used, but with 6 well plates and a seeding density of 1 x 10^6^ cells/well, and in the absence or presence of 100 nM (final concentration) AEA.

### LPS + INFγ treatment of RAW264.7 cells

Cells were cultured in 75cm^2^ flasks at 37°C with 5% CO_2_ at humidified atmospheric pressure and split (ratio 1:4–8) approximately twice a week. Cells (2x10^5^/well) were seeded into 24 well plates and allowed to attach and equilibrate over night. The next day the cells where exposed to 0.1 μg ml^-1^ LPS and 100U ml^-1^ IFNγ (final concentrations) or vehicle (medium containing 1 μM NaHPO_4_ pH 8.0 supplemented with 10^−5^% bovine serum albumin (BSA) w/v, final concentrations) for 24h. This treatment produces a large induction of COX-2 in the RAW264.7 cells (see e.g. [31]).

### mRNA extraction and qPCR of the DU145 and RAW264.7 cells

After treatment, the wells were washed with PBS followed by addition of 300 μL of lysis/binding buffer (Thermo Fisher Scientific, Waltham, MA) and the plates where stored in -80°C for later assessment. mRNA was extracted using DYNABEADS® mRNA DIRECT™ purification kit followed by cDNA convertion using High-Capacity cDNA Reverse Transcription Kit by Thermo-Fisher Scientific. The cDNA was diluted 1:10 before real-time quantitative PCR was performed using KAPA SYBR FAST qPCR Master Mix. An Illumina® ECO™ Real-Time PCR system was used with the ECO™ Software v4.0.7.0. Data are normalized to the mRNA expression of the 60S ribosomal protein L19 (RPL19). Primer sequences are given in Table 1. The efficiencies of all primer pairs were determined, and were between 92–108%. Ct values were left uncorrected. Data are presented as ΔCt (i.e. Ct for gene of interest–corresponding Ct of RPL19). In Table 2, the values for the treated samples as a % of the values for control samples at the same time point are also shown, calculated using the 2-ΔΔCt method.

**Table 2:** Effect of treatment of DU145 cells with TNFα upon mRNA levels of AEA and 2-AG metabolic enzymes.

	Control			TNFα			TNF as		
		95% CI			95% CI		% of C		
Time (h)	Mean	Lower	Upper	Mean	Lower	Upper	(2-ddCt)	P values	
*NAPEPLD*									
0	7.95	7.74	8.15	8.19	7.78	8.61	84	TNFα	0.0003
1	9.18	8.72	9.65	9.04	8.89	9.20	110	Time	<0.0001
2	8.99	8.72	9.25	9.58	9.22	9.95	66	TNFα x Time	0.023
3	8.37	8.19	8.56	8.67	8.43	8.91	81		
4	8.14	7.93	8.34	8.56	8.38	8.75	74		
*FAAH*									
0	8.86	8.55	9.17	8.97	8.72	9.22	93	TNFα	0.18
1	9.11	8.92	9.30	9.32	8.93	9.71	86	Time	<0.0001
2	9.31	8.94	9.68	9.59	9.23	9.94	82	TNFα x Time	0.47
3	9.44	9.30	9.57	9.37	9.19	9.54	105		
4	9.27	9.10	9.44	9.26	8.98	9.54	101		
*NAAA*									
0	7.18	6.78	7.58	7.40	6.95	7.84	86	TNFα	0.67
1	7.42	7.14	7.69	7.68	7.29	8.08	83	Time	0.091
2	7.42	6.88	7.96	7.60	7.25	7.95	88	TNFα x Time	0.20
3	7.23	6.84	7.63	7.20	6.96	7.44	102		
4	7.77	7.34	8.20	7.35	7.00	7.70	134		
*PTGS1*									
0	13.63	12.84	14.42	13.04	12.46	13.62	150	TNFα	0.038
1	12.93	12.61	13.24	13.72	13.09	14.35	58	Time	0.0002
2	14.28	13.72	14.85	14.40	13.45	15.35	92	TNFα x Time	0.027
3	13.04	12.80	13.29	13.57	12.18	14.96	69		
4	13.51	13.02	14.00	14.79	14.33	15.25	41		
*PTGS2*									
0	13.65	13.12	14.18	13.40	12.89	13.91	119	TNFα	<0.0001
1	11.95	11.18	12.73	9.96	9.60	10.32	397	Time	<0.0001
2	13.26	12.80	13.73	11.51	11.19	11.84	336	TNFα x Time	<0.0001
3	13.25	12.96	13.55	11.10	10.81	11.39	446		
4	13.77	13.11	14.42	11.90	11.67	12.13	364		
*cPLA2g4a*									
0	11.21	10.85	11.57	11.41	10.71	12.11	87	TNF	0.94
1	11.34	11.06	11.62	11.51	11.24	11.79	89	Time	0.0048
2	11.60	11.26	11.95	11.55	11.07	12.02	104	TNF:Time	0.91
3	11.57	11.22	11.93	11.28	10.22	12.34	122		
4	12.10	11.78	12.42	11.95	11.76	12.14	111		
*DAGLa*									
0	8.72	8.50	8.94	8.85	8.55	9.16	91	TNF	<0.0001
1	9.31	9.00	9.62	9.25	8.87	9.63	104	Time	<0.0001
2	9.29	8.97	9.60	10.06	9.63	10.50	59	TNF:Time	<0.0001
3	9.32	9.04	9.59	10.58	10.14	11.02	42		
4	9.11	8.74	9.48	10.66	10.39	10.92	34		
*DAGLb*									
0	8.49	8.22	8.76	8.42	8.26	8.58	105	TNF	<0.0001
1	8.57	8.35	8.79	8.73	8.31	9.16	89	Time	<0.0001
2	8.73	8.48	8.98	9.50	9.22	9.79	58	TNF:Time	<0.0001
3	8.78	8.51	9.06	9.62	9.33	9.91	56		
4	8.80	8.52	9.08	9.60	9.39	9.82	57		
*MGLL*									
0	5.13	4.97	5.29	5.31	5.09	5.53	88	TNF	1.00
1	5.22	4.92	5.53	5.14	4.80	5.49	105	Time	0.35
2	5.11	4.89	5.33	5.30	4.80	5.81	88	TNF:Time	0.30
3	5.04	4.83	5.24	5.01	4.76	5.26	102		
4	5.21	5.09	5.33	5.07	4.85	5.29	110		
*ABHD6*									
0	6.94	6.69	7.19	7.21	6.53	7.89	83	TNF	0.039
1	7.34	7.11	7.57	7.65	6.98	8.31	81	Time	0.0004
2	7.48	7.14	7.82	8.21	7.72	8.70	60	TNF:Time	0.35
3	6.97	6.31	7.62	6.88	6.08	7.68	106		
4	7.16	6.46	7.86	7.36	7.04	7.67	87		
*ABHD12*									
0	5.41	4.94	5.87	5.77	5.26	6.28	78	TNF	0.62
1	5.56	5.27	5.85	5.97	5.29	6.66	75	Time	0.18
2	5.69	5.43	5.95	6.13	5.36	6.90	74	TNF:Time	0.063
3	5.79	5.30	6.28	5.27	4.36	6.18	143		
4	6.15	5.52	6.79	5.85	5.47	6.23	124		

DU-145 cells were treated for the times shown with vehicle or 20 ng mL^-1^ TNFα prior to lysis of cells and extraction of mRNA. Shown are means and 95% confidence limits of the ΔCt values for 5–6 separate lysates produced on the same experimental day. A change in the ΔCt value of 1 unit represents a two-fold change in the mRNA level. To aid the reader, the mean value for the TNFα-treated samples as % of the mean control values at the same time point, calculated using the standard 2-ΔΔCt method, are shown in the table. Unadjusted P values were determined for the ΔCt data by permutation test using the function lmp followed by Anova in the lmPerm package for R. At a 5% false discovery rate [42], the critical P value is 0.024.

### Western blot for COX-2

DU145 and RAW264.7 cells were plated in T75 culture flasks and COX-2 was induced as described above. After COX-2 induction, the cells were washed once in PBS before being collected by scraping with a rubber policeman. Cells where pelleted (5min, 90 x g, 4° C) and lysed in lysis buffer (150 mM NaCl, 1% Triton X100, 50mM Tris buffer, pH 8.0 with protease inhibitor). Sonication was used further to disrupt the cells before debris was removed by centrifugation (5min, 14000*g*, 4°C). The protein content of the supernatant was determined using a Pierce™ BCA Protein Assay kit. Samples were stored in -80°C until mixed in 5x Laemmli sample buffer, thereafter stored in -20°C.Aliquots (50–210 μg protein) were loaded on BIO-RAD mini-protean TGX Stain-free™ precast gels (4–20%) and separated at 100V. Human recombinant COX-2 (10 ng) was loaded as positive control and the BIORAD precision Plus unstained standard ladder was applied on both ends of the gel. Proteins where blotted on to mini PVDF membranes (0.2 μm) using the Trans Blot Turbo Transfer system from BIO-RAD, 30 min 25V, 1.0A. Efficiency of protein separation and blotting was monitored using the Chemidoc MP system from BIO-RAD. Membranes where blocked in TBST (500 mM NaCl, 0.1% Tween-20, 20 mM Tris, pH 7.4) with 5% dry milk for 1h. They were then incubated over night at 4°C with the primary polyclonal antibody for COX-2 (rabbit anti-mouse, cat #: 160106; Cayman Chemical Co.). The next day the membrane was washed 6 x 5min in TBST and incubated for 1 h at room temperature with HRP-conjugated secondary antibody (polyclonal goat anti-rabbit immunoglobulin/HRP, Dako, Glostrup, Denmark). The membranes were washed again 6 times 5 min in TBST, incubated for 2 min in electrochemiluminescence fluid and signals where detected using the BIO-RAD Chemidoc MP system, exposure time 1–300 s, 10 capture moments.

### Anandamide uptake and hydrolysis

The uptake of exogenous AEA in the DU145 and RAW264.7 cells was measured by a method developed by Rakhshan et al. [32] as modified by Sandberg and Fowler [33]. Following the treatment paradigms described above, the cells were washed twice with 400 μL of pre-warmed KRH buffer (120 mM NaCl, 4.7 mM KCl, 2.2 mM CaCl_2_, 10 mM 4-(2-hydroxyethyl)-piperazineethane-sulfonic acid (HEPES), 0.12 mM KH_2_PO_4_, 0.12 mM MgSO_4_, pH 7.4) with and without 1% BSA. Thereafter, 340 μL of pre-warmed KRH buffer with 0.1% fatty acid-free BSA and 10 μL of test compound or vehicle (final solvent concentration was 0.2% DMSO) was added and the cells were incubated for 10 min at 37°C. After the preincubation phase, [Ara-^3^H]AEA (50 μL, final concentration 100 nM, 0.4 μCi mL^-1^, in KRH buffer with 0.1% fatty acid-free BSA) was added and the cells were incubated for a further 5–30 min at 37°C. Uptake was stopped by addition of 600 μL of 0.2 M NaOH. The content of each well was mixed and aliquots (300 μL) were transfered to scintillation vials together with 4 mL scintillation fluid and analysed for tritium content by liquid scintillation with quench correction. Retention of the ligand by the plastic wells was assessed concomitantly using the same assay but in the absence of cells.

[^3^H]AEA hydrolysis by the cells was measured using the same protocol as above but with [Et-^3^H]AEA as substrate (100 nM final concentration), and with a different work-up [34]. Thus, after the incubation phase, the hydrolysis was stopped by addition of 600 μL of activated charcoal solution (120 μL activated charcoal + 480 μL 0.5 M HCl). The content of each well was mixed and 600 μL was transferred to glassware that were centrifuged at 1200 *g* for 10 min at room temperature. Aliquots (200 μL) of the aqueous phase were transferred to scintillation vials together with 4 mL scintillation fluid and analysed for tritium content by liquid scintillation with quench correction. Blanks were wells without cells.

Additional parallel plates where included in each experiment for measurement of protein content using Pierce™ BCA protein assay kit and for confirmatory mRNA measurements (described above).

### Thin-Layer chromatography

DU145 cells (3.5 x 10^5^ cells/well) and RAW264.7 cells (4 x 10^5^/well) were plated in 12 well plates and treated with TNFα for 4 h or LPS/IFNγ for 24 h, respectively, as described above. [Ara-^3^H]AEA (100 nM final concentration, 0.4 μCi mL^-1^ in FBS containing medium) was then added to the wells. After 30 min of incubation at 37°C, the medium (800μL) was collected and the cells scraped using a rubber policeman into 200 μL PBS. Lipids were extracted by the method of Bligh and Dyer [35]. Briefly 800 μL chloroform and methanol (1:2) was added and the samples were mixed for 15 min on an orbital shaker. After addition and mixing with an additional 200 μL chloroform and 200 μL milliQ water, the samples were centrifuged at 1000*g* x 5 min to separate the phases. Aliquots (25 μL) of the chloroform phases were applied to a 5 x 20 cm silica TLC plate (Partisil LK5D, Silica Gel 150 Å, layer thickness 250 μm; Whatman International Ltd, Maidstone UK). Lipids were separated in 90% Ethyl acetate, 10% methanol [36] (total separation length was 16.25 cm). The plates where dried at 100°C for 5 min and 7.5 mm strips of each lane was scraped into scintillation vials and analysed for tritium content by liquid scintillation with quench correction. In parallel experiments, cell extracts were spiked with the standard compounds ([^3^H]AA, [Ara-^3^H]AEA, [^3^H]PGF_2α_ and [^3^H]bimatoprost (as a marker for PG-EAs) and added to silica plates as described above in order to establish their retention factors (R_f_). Data are expressed as % of the tritium recovered with respect to the corresponding tritium content in the chloroform extract.

### Measurement of AEA, related NAEs, PG-EA, PGs and 2-AG in DU145 cells by ultra-performance liquid chromatography-tandem mass spectrometric (UPLC–MS/MS) analysis

DU145 cells were treated with TNFα for 0–4 h as described above. In one experimental setup the treatment was followed by addition of AEA (100 nM final concentration) to each well and continued incubation for 2h. Aliquots (200 μL) of medium were taken at each indicated time point. After incubation, the plates were placed on ice, the medium was aspirated, and the cells were washed twice with ice-cold PBS (2 x 1 mL). One mL of methanol was added to the wells, the mixture was scraped using a rubber policeman and the extract pipetted into Falcon tubes. An additional 1 ml of methanol was added to the wells, the wells scraped and the mixture was pipetted into the same tubes. These were centrifuged (2000 × g for 15 min) to sediment cell debris, and the methanol phase was collected and stored at -80°C until analysis.

The methanolic extracts were diluted with milliQ water to a final concentration of 5% methanol (v/v). After samples were spiked with 10 μL of internal standards solutions (deuterated lipids, of which those relevant for the present study are 40 ng mL^-1^ PGE_2_-EA-d_4_, 20 ng mL^-1^ AEA-d_4_, PEA-d4, and OEA-d_4_, 800 ng/mL 2-AG-d_8_, 25 ng mL^-1^ PGE_2_-d_4_, PGD_2_-d_4_, TXB_2_-d_4_ and 20-HETE-d_6_), 10 μL antioxidant solution (0.2 mg mL^-1^ BHT/EDTA in methanol/water (1:1)) and then applied directly to Waters Oasis HLB SPE cartridges (200 mg of sorbent, 30 μm particle size) (for details of the complete internal standards mixture, see the original methodology paper of Gouveia-Figueira and Nording [37]). Cartridges were washed with 2 mL of ethyl acetate, followed by 2x2 mL of MeOH, and then conditioned with 2x4 mL of wash solution (WS, 95:5 v/v water/MeOH with 0.1% acetic acid). After loading the sample containing internal standard and antioxidant solutions, the cartridges were washed with 2x4 mL of WS, and eluted with 4 mL acetonitrile, followed by 2 mL of MeOH and 1 mL of ethyl acetate. Eluates were concentrated with a MiniVac system (Farmingdale, NY, U.S.A.) and reconstituted in 100 μL of MeOH and vortexed. Solutions were then transferred to LC vials with low-volume inserts, 10 μL of CUDA (50 ng mL^-1^) was added, and analysis was performed immediately using an Agilent ultra-performance (UP)LC system (Infinity 1290) coupled with an electrospray ionization source (ESI) to an Agilent 6490 Triple Quadrupole system equipped with the iFunnel Technology (Agilent Technologies, Santa Clara, CA, USA). [35]

Chromatographic separation of the analytes was performed with separate injections for subsequent ionization in positive (AEA, OEA, PEA, LEA, PGE_2_EA, 2-AG) and negative (PGD_2_, PGE_2_, TXB_2_ and 15-HETE) mode. Analyte separation was performed using a Waters BEH C18 column (2.1 mm x 150 mm, 2.5 μm particle size), and 10 μL injection volumes were employed for each run. The mobile phase consisted of (A) 0.1% acetic acid in MilliQ water and (B) acetonitrile:isopropanol (90:10). The following gradients were employed: positive mode, 0–2.0 min 30–45% B, 2.0–2.5 min 45–79% B, 2.5–11.5 min 79% B, 11.5–12 min 79–90% B, 12–14 min 90% B, 14–14.5 min 90–79% B, 14.5–15.5 min 79% B, 15.6–19 min 30% B; negative mode, 0–3.5 min 10–35% B, 3.5–5.5 min 40% B, 5.5–7.0 min 42%B, 7.0–9.0 min 50% B, 9.0–15.0 min 65% B, 15.0–17.0 min 75% B, 17.0–18.5 min 85% B, 18.5–19.5 min 95% B, 19.5–21 min 95–10% B, 21.0–25.0 min 10% B. Precursor ions, [M+H]^+^ and [M-H]^-^, product ions, multiple reaction monitoring (MRM) transitions and optimal collision energies were established for each analyte. ESI conditions were: capillary and nozzle voltage at 4000 V and 1500 V, drying gas temperature 230°C with a gas flow of 15 L min^-1^, sheet gas temperature 400°C with a gas flow of 11 L min^-1^, the nebulizer gas flow was 35 psi, and iFunnel high and low pressure RF at 90 and 60 V (negative mode) and 150 and 60 V (positive mode). The dynamic MRM option was performed for all compounds with optimized transitions and collision energies. The MassHunter Workstation software was used to manually integrate all peaks. For further details, see [37].

### Statistics

For the mRNA data, Fisher’s randomization (permutation) tests were used with the R statistical program, versions 3.3.1 and 3.3.2 [38] in place of standard parametric tests since they make fewer assumptions about the dataset (for a description of these approaches, see [39]). The different permutation tests used were permTS in the perm package for comparison of two independent variables and lmp followed by Anova in the lmPerm package for two-way analyses. In all cases, at least 10000 iterations were used. For the uptake and hydrolysis experiments, a bootstrapped linear model [40] was used in preference to a three-way ANOVA in view of the differences in variances between the groups. The R code for this model is available in [40]. Additionally, one-way ANOVA not assuming equal variances were calculated using GraphPad Prism v7 for the Macintsh (GraphPad Software Inc., San Diego, CA, USA). For the lipidomics data, where there are repeated measures but occasional missing values, linear mixed models (function lme in the package nlme for R) were used where the factors are added sequentially, as described by Field et al. [41]. When multiple P values were generated, critical P values were calculated using a 5% false discovery rate [42].

## Results

### Effects of TNFα treatment upon the mRNA levels of AEA and 2-AG synthetic and catabolic enzymes in DU145 cells

**Fig 1:**
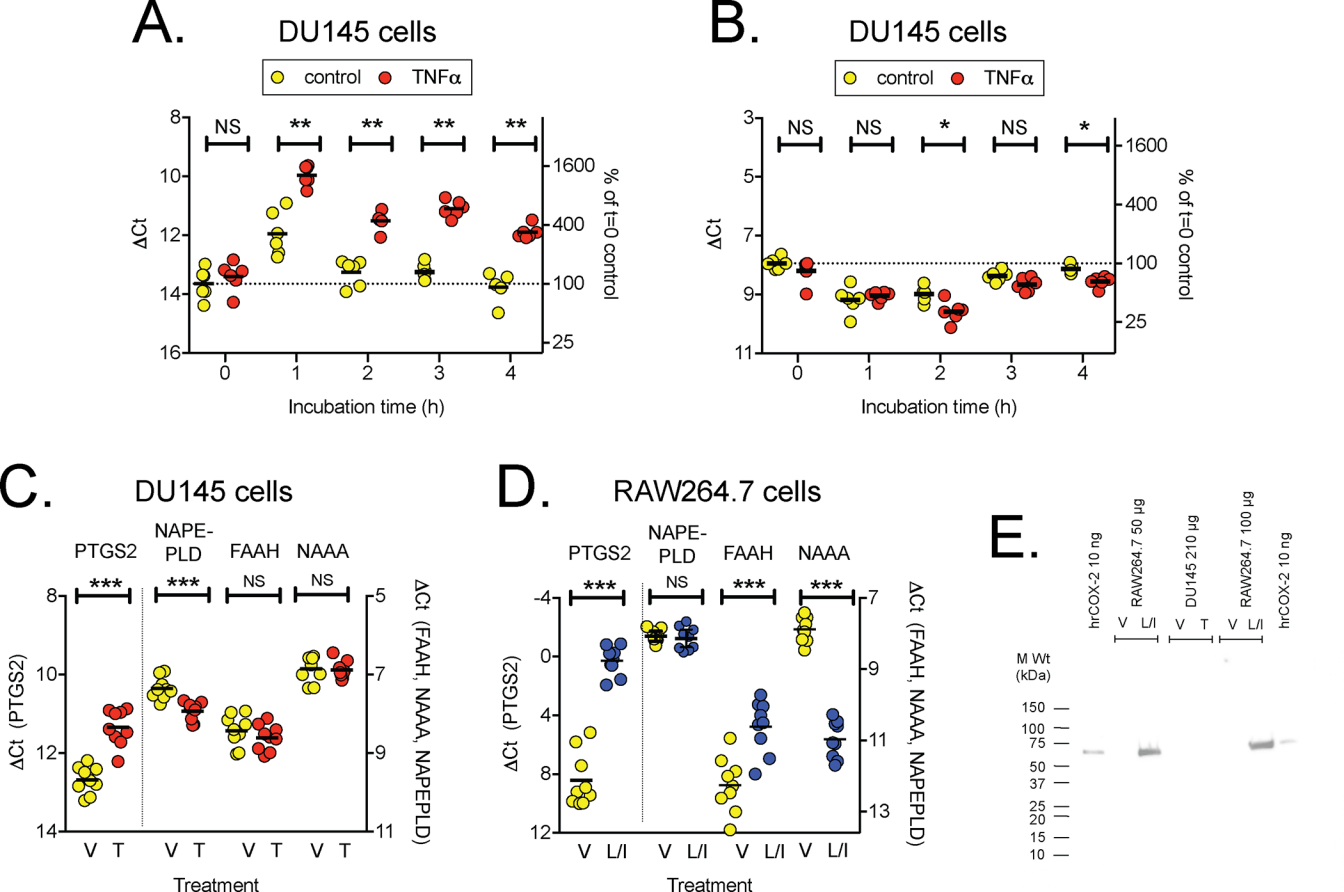
Expression of *PTGS2* (COX2), *NAPEPLD*, *FAAH* and *NAAA* in DU145 and RAW264.7 cells. Panels A and B. mRNA levels of *PTGS2* (A) and *NAPEPLD* (B) measured in DU145 cell lysates at different times after TNFα treatment. The individual data points summarised in Table 2 are shown for these two proteins. Panels C and D show mRNA for *PTGS2*, *NAPEPLD*, *FAAH* and *NAAA* in control (V) and TNFα treated (2 h, T) DU145 cells (Panel B), and in control and LPS + IFNγ-treated (24 h, L/I) RAW264.7 cells (Panel C). Data are for 8–9 separate experiments, undertaken concomitantly with the uptake experiments shown in Fig 2. ***P<0.001, **P<0.01, ^NS^P>0.05, exact two-sided permutation tests (complete enumeration) for the comparison shown. Panel E shows a Western blot for COX-2 with unstimulated and stimulated DU145 (TNFα) and RAW264.7 (LPS + IFNγ) cells. Human recombinant COX-2 is included as a positive control.

In the first experiment, DU145 cells were treated with 20 ng ml^-1^ of TNFα for 1–4 h prior to measurement of the mRNA levels of these proteins. Data, as ΔCt with RPL19 as housekeeping gene, are shown in Table 2, and, for illustrative purposes, the changes in *PTGS2* and *NAPEPLD* in Fig 1A and 1B. A decrease in the ΔCt value of 1 unit represents a doubling of the mRNA level. Consistent with the literature [30], TNFα treatment of the cells increased the expression of *PTGS2*, and a significant increase was already seen at 1 h following treatment. In contrast, a decrease in *NAPEPLD* expression was seen, whereas the cytokine was without effects upon *FAAH* and *NAAA* expression. These results were confirmed at the 4 h time-point using samples from nine separate experiments run concomitantly with the uptake experiments described below (Fig 1C). It was noted that the expression levels of *PTGS2* were relatively low in the cells, even following TNFα treatment. This is in contrast to the very high levels seen in RAW264.7 macrophages following induction by LPS + IFNγ treatment, a treatment that also increases *Faah* and reduces *Naaa* expression (Fig 1D), making these cells a useful positive control, albeit from a different species. Western Blot was used to investigate translation of *PTGS2* mRNA into COX2 protein. A COX-2 immunoreactive band was seen for LPS + IFNγ treated RAW264.7 cells, but not for the unstimulated cells (Fig 1E). Given that the expression level of *PTGS2* in the DU145 cells is lower than the corresponding *Ptgs2* for the unstimulated RAW264.7 cells (compare Fig 1C and 1D), it is not surprising that the Western blot was not sensitive enough to detect COX-2 immunoreactivity of either vehicle or TNFα-treated DU145 cells (Fig 1E).

Although the focus of this manuscript was upon AEA, we also measured mRNA for enzymes involved in the synthesis and catabolism of 2-AG, since this endocannabinoid plays an important role in the invasivity of prostate cancer cells [14,43,44]. TNFα treatment of the cells reduced the levels of the 2-AG synthetic enzymes *DAGLa* (diacylglycerol lipase α) and *DAGLb* after ≥2 h of treatment, whereas there was no significant effect of the TNFα treatment, or the interaction TNFα x time, upon the levels of the hydrolytic enzymes *MGLL* (monoacylglycerol lipase), *ABHD6* (abhydrolase domain containing 6) or *ABHD12* (Table 2). It was noted that the mRNA levels for *MGLL* and *ABHD12* were considerably higher than for *FAAH*, *NAAA* or *PTGS2*.

### Accumulation of [Ara-^3^H]AEA by unstimulated and stimulated DU145 and RAW264.7 cells: Role of COX-2

**Fig 2:**
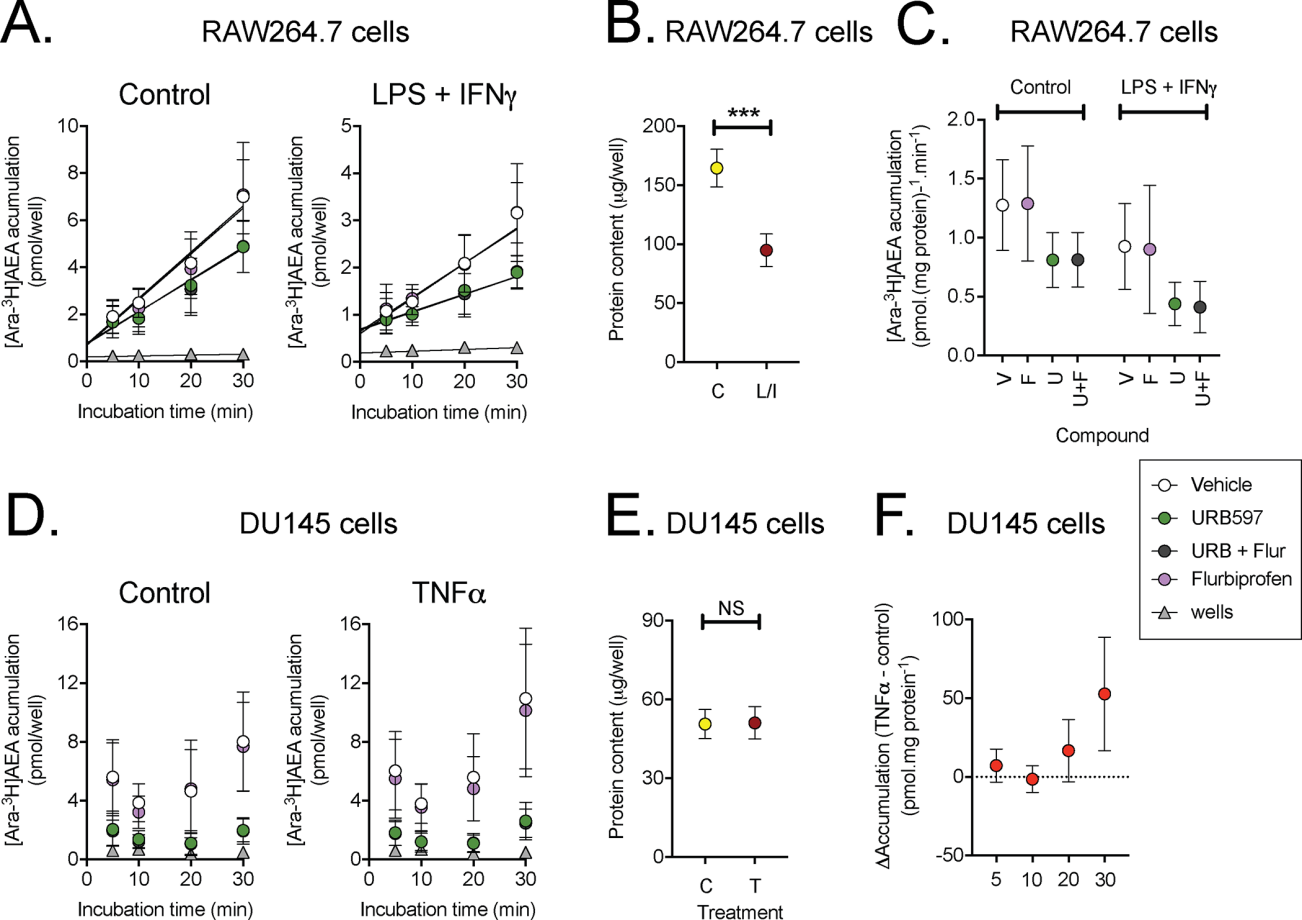
Uptake of 100 nM [Ara-^3^H]AEA into RAW264.7 and DU145 cells. Panel A-C show data for control and LPS + IFNγ-treated RAW264.7 cells; Panels D-F for control and TNFα-treated DU145 cells. Panels A, D show the time courses for the accumulation of radiolabel 5, 10, 20 and 30 min after addition of 100 nM of [Ara-^3^H]AEA. Note that the uptake is per well, and not normalised to the protein content (shown in Panels B and E). In Panel C, rates of uptake were determined for each experiment from the individual slopes of each the time course divided by the protein content. Shown are means and 95% confidence intervals, N = 8. Data was analysed using bootstrapped linear models (for details, see [40]). For the main effects model, the P values were: LPS + IFNγ, P<0.0001; flurbiprofen, P = 0.89; URB597, P<0.0001. For the interactions model, the P values of the three bivariate interactions and the trivariate interaction were all ~0.9. The time courses for uptake into DU145 cells shown in Panel D could not be used to obtain robust slope replots. In consequence, the difference in uptake (per unit protein) between TNFα-treated and control cells were determined for each time point. The data is shown in Panel F (means and 95% confidence intervals, N = 6–7). A one-way ANOVA not assuming equal variances gave a P value of 0.018. Note that at the 30 min incubation time point, the confidence limits do not straddle zero.

The expression level of COX2 in the DU145 cells is too low to determine whether the enzyme regulates the cellular uptake of AEA analogous to the situation for FAAH [8], but the high levels of COX-2 in the LPS + FNγ-treated RAW264.7 cells make them ideal in this respect. Time courses of 100 nM [Ara-^3^H]AEA accumulation by control and LPS + IFNγ-treated RAW264.7 cells are shown in Fig 2A. The treatment reduced the protein concentration in the cells (Fig 2B), which is a complicating factor, but from the time course data, rates of accumulation per mg protein could be calculated (Fig 2C). Inhibition of FAAH by the selective inhibitor URB597 [45] reduced, as expected, [Ara-^3^H]AEA accumulation, whereas the COX inhibitor flurbiprofen, at a concentration that totally blocks PG production by the cells [31] was without significant effect upon the AEA accumulation. When expressed as % of the corresponding vehicle value for the same condition, the means (95% CI), N = 8 for flurbiprofen were: control, 101 (87–115); LPS + IFNγ-treated, 92 (71–113). These data indicate that, in contrast to FAAH, COX-2 does not regulate the uptake of [Ara-^3^H]AEA into RAW264.7 cells under the conditions investigated.

The uptake of 100 nM [Ara-^3^H]AEA by the DU145 cells is shown in Fig 2D–2F. In this case, the TNFα treatment did not affect the total protein concentration of the cells. However, the time-course data were not sufficiently robust to determine rates of accumulation, although it was clear that URB597 reduced the uptake of [Ara-^3^H]AEA. In consequence, we compared the difference between the uptake (normalised for protein) for TNFα-treated cells and control cells at each incubation time. At the 30 min time point, but not at the other time points, the accumulation was greater in the TNFα-treated cells than in the control cells (Fig 2F).

### Hydrolysis of [Et-^3^H]AEA by control and TNFα-treated DU145 cells

**Fig 3:**
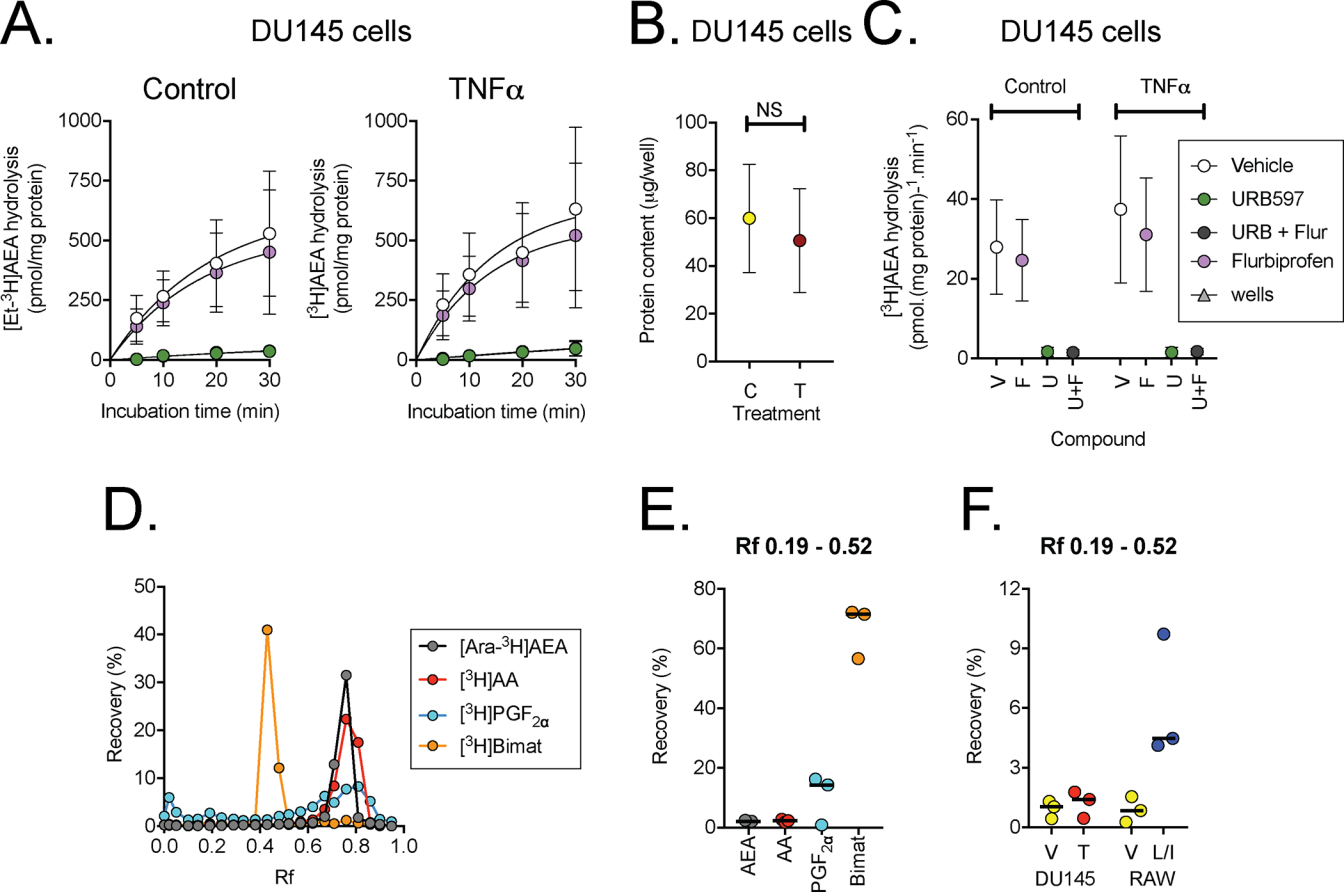
Metabolism of 100 nM AEA by DU145 cells. Panels A show the time courses for the hydrolysis of 100 nM [Et-^3^H]AEA. Note that the values are per well, and not normalised to the protein content (shown in Panel B). In Panel C, rates of hydrolysis were determined for each experiment from the individual slopes (going through the origin) of the first two time points divided by the protein content. Data are means and 95% confidence intervals, N = 9. For the vehicle and flurbiprofen treated cells, the bootstrapped linear main effects model gave P values of TNFα, 0.19; flurbiprofen, 0.62. The interaction model gave a P value for TNFα x flurbiprofen of 0.80. However, a small effect of flurbiprofen can be masked by the large inter-experimental variation. Expressing the effect of flurbiprofen as % of the corresponding control value gave values of: untreated cells, 90 (81–99.5); TNFα- treated cells 87 (77–96) (means and 95% confidence limits, N = 9). Panels D-E: TLC separation of [Ara-^3^H]AEA, [^3^H]arachidonic acid (AA), [^3^H]PGF_2α_ and [^3^H]bimtoprost (Bimat) using ethyl acetate: methanol (90:10 v/v) as solvent system. Panel D shows the complete sampling from a single experiment, and Panel E shows the total recovery for three separate experiments over the R_f_ range shown. In Panel F, cells were incubated with 100 nM [^3^H]AEA, labelled in the arachidonoyl part of the molecule for 30 min prior to workup and separation by TLC. Shown are means of individual experiments conducted in triplicate for vehicle (V) and TNFα (T)-treated DU145 cells, and for vehicle and LPS + IFNγ-treated (L/I) RAW264.7 cells.

Time courses of the hydrolysis of 100 nM [Et-^3^H]AEA by DU145 cells are shown in Fig 3A, and the protein content in Fig 3B. The hydrolysis of [Et-^3^H]AEA was essentially linear over the first ten minutes of incubation, these were used to calculate the initial rates of hydrolysis shown in Fig 3C. As expected, the FAAH inhibitor URB597 produced complete inhibition of the hydrolysis. A very small (~10%, see legend to figure) inhibition of hydrolysis was seen with 10 μM flurbiprofen, consistent with the modest FAAH-inhibitory properties of this compound (IC_50_ value 29 μM in rat brain homogenates [46]) but insufficient to affect AEA uptake due to this mechanism (Fig 2D). There was no significant effect of TNFα treatment upon the observed rate of [Et-^3^H]AEA hydrolysis by the cells (Fig 3C).

### Separation of lipid products by thin layer chromatography after incubation of DU145 and RAW264.7 cells with [Ara-^3^H]AEA

Cells were incubated for 30 min with 100 nM [Ara-^3^H]AEA, after which lipid extracts were separated using a 90% ethyl acetate, 10% methanol solvent system. In this system, PGE_2_-EA has a lower R_f_ value than either AEA or PGE_2_ [33]. In our hands, [Ara-^3^H]AEA, [^3^H]PGF_2α_, and [^3^H]arachidonic acid all eluted at around the same R_f_ (~0.7–0.9), whereas the PG-EA analogue [^3^H]bimatoprost (17-phenyl trinor prostaglandin F_2α_ ethyl amide) was eluted at a lower R_f_ (~0.4–0.65) (Fig 3D and 3E). This is consistent with the study of [36], although the R_f_ values for all of the compounds in that study were lower than ours, possibly due to the use of a different TLC plate (60 Å, as compared with 150 Å here). Following incubation with [Ara-^3^H]AEA (100 nM), recovery of tritium in the Rf 0.19–0.52 range was very low in the preliminary experiments undertaken with either vehicle or TNFα-treated DU145 cells or with the vehicle-treated RAW264.7 cells. However, a higher recovery was seen in the LPS+IFNγ-treated RAW264.7 cells (Fig 3F). This would suggest that at high levels of COX-2 expression, treatment of RAW264.7 cells with AEA results in sufficient PG-EA production to be measureable by TLC, whereas at the low COX-2 expression levels in DU145 cells, insufficient PG-EA formation occurs to be detectable by this method.

### Measurement of AEA, related NAEs, PG-EA, PGs and 2-AG in DU145 cells by UPLC–MS/MS

**Fig 4:**
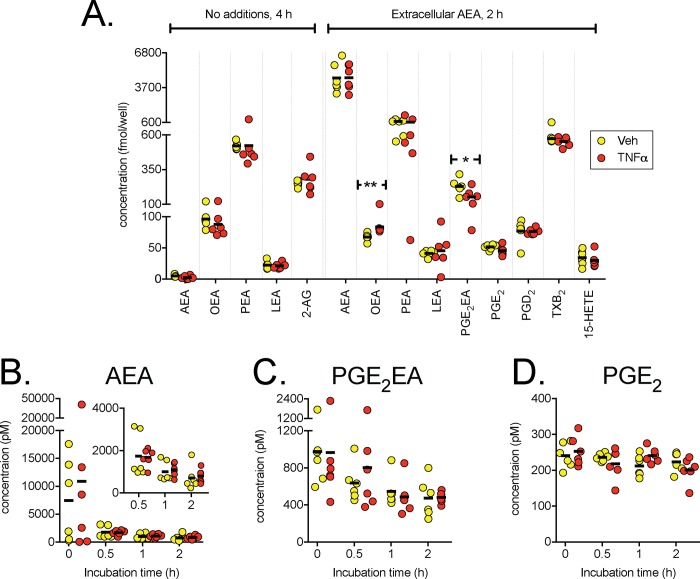
*N*-acylethanolamine, 2-AG and oxylipin levels in TNFα-treated DU145 cells. Panel A, levels of lipids extracted from DU145 cells after treatment with vehicle or TNFα. AEA (100 nM) was added to the medium for the samples indicated as “Extracellular AEA”. Individual data points are shown, with the bars indicating the mean values. *P = 0.046, **P = 0.0022, two-tailed exact permutation test, otherwise P>0.05. At a 5% false discovery rate [40], the critical P value is 0.0036. Panels B-D show lipid concentrations in aliquots of the medium after addition of AEA taken at different times after incubation with vehicle or TNFα. The statistical treatment of the data for PGE_2_EA and PGE_2_ is given in Table 4. For AEA, the very large difference in variance between the first and the subsequent time points is an issue, but analysis of the log-transformed data for the 0.5, 1 and 2 h time points (shown with a smaller scale in the insert to Panel B) gave P values of time <0.0001, TNFα 0.47, interaction 0.76, with acceptable residual plots.

In order to determine whether PG-EA formation occurs in the TNFα-treated DU145 cells, we used a highly sensitive UPLC-MS/MS system for analysis of cell and medium extracts [37]. Two experiments were undertaken. In the first, the cells were treated with vehicle or TNFα, and aliquots of the medium were taken up to 4 h of incubation. Data were analysed using a linear mixed model where the influence of each factor (time, TNFα treatment and time x TNFα treatment) added sequentially is determined [41]. AEA levels were extremely low, and there was no significant improvement of the linear model containing time as a factor when TNFα was added to the model (Table 3). PEA, OEA and LEA were also quantified, with a similar lack of effect of TNFα. There was an improvement in the model for PEA when the interaction term time x TNFα was added. For the cell extracts, none of the NAEs showed significant differences in levels between control and TNFα-treated cells (Fig 4A). No PG or PG-EA species were detected in either the samples of the medium or the cell extracts.

**Table 3:** Concentrations (pM) of AEA, PE, OEA, LEA and 2-AG in the medium following TNFα treatment of DU145 cells.

	Control			TNFα				
Lipid Time(h)	Mean	min	max	mean	min	max	lme ANOVA	P
AEA								
0	0	0	0	8.7	0	15	Time	0.0001
1	4.0	0	20	0	0	0	Time+TNFα	0.69
2	11	8.1	12	7.2	0	19	Interaction	0.026
3	6.5	0	17	11	0	16		
4	13	8.9	17	14	8.9	19		
PEA								
0	512	397	656	458	403	563	Time	0.0001
1	726	572	885	513	405	612	Time+TNFα	0.95
2	533	439	610	622	439	761	Interaction	<0.0001
3	462	360	531	473	340	559		
4	297*	273	319	475	401	545		
OEA								
0	72	55	94	79	52	163	Time	0.32
1	80	72	95	73	58	106	Time+TNFα	0.29
2	60	52	68	81	67	115	Interaction	0.47
3	66	50	81	69	7.1	96		
4	55*	52	59	66	55	86		
LEA								
0	81	74	91	73	51	113	Time	0.0068
1	93	82	106	86	78	108	Time+TNFα	0.97
2	97	79	109	98	79	116	Interaction	0.44
3	80	66	97	81	54	104		
4	65	62	67	85	64	107		
2-AG								
0	417	357	480	408	257	526	Time	0.041
1	423	325	596	425	284	637	Time+TNFα	0.68
2	399	298	504	385	268	587	Interaction	0.18
3	438	387	521	455	380	533		
4	415*	319	524	522	347	790		

Values are means with ranges of the concentrations for aliquots taken at the time points shown. A linear mixed model (function lme in the package nlme for R) was used as described by Field et al. [41], where the factors are added sequentially. The P values are for the ANOVA of the baseline, Time, Time+TNFα and interaction models. At a 5% false discovery rate [42], the critical P value was 0.013. Residual plots were deemed to be acceptable in all cases. Sample sizes were n = 6 for TNFα and n = 5 for controls.

*For these cases, one sample was excluded, since the values were clearly anomalous (PEA 3520 pM; OEA 1008 pM; 2-AG 1789 pM).

In the second experiment, 100 nM AEA was added to the medium with the TNFα (or vehicle) after 4h of incubation and aliquots of the medium were collected over the next 2 h. AEA was rapidly cleared from the medium (Fig 3B), and PGE_2_-EA, PGD_2_, PGE_2_, TXB_2_ and 15-HETE were detected in both medium and cell extracts in addition to the NAEs (Fig 3A, 3C and 3D and Table 4). PGE_2_-EA was also cleared from the medium, albeit more slowly than AEA (compare Fig 3B and 3C). With the exception of 15-HETE, there was no improvement in the linear model with time as a factor by the addition of TNFα treatment to the model, although addition of the interaction term did cause further improvements in the case of PGD_2_, TXB_2_ and 15-HETE (Table 4). For the cell lysates, the only significant change upon implementation of a 5% false discovery rate was for OEA, which was higher in the TNFα-treated cells (Fig 4A). These data would suggest that the DU145 cells produce measureable levels of NAEs per se, but not of PGs and PG-EAs, unless AEA is provided exogenously.

**Table 4:** Concentrations (pM) of lipids in the medium following treatment with TNFα and addition of AEA to DU145 cells.

	Control			TNFα				
Lipid Time (h)	Mean	min	max	mean	min	max	P value	
PEA^†^								
0	755	627	889	847	695	1015	Time	0.037
0.5	621	499	1023	656	585	734	Time+TNFα	0.46
1	635	469	771	580	450	683	Interaction	0.10
2	877	506	2373	530	459	612		
OEA^†^								
0	88	64	110	97	77	137	Time	0.0016
0.5	116	98	136	95	79	104	Time+TNFα	0.12
1	119	97	132	105	81	144	Interaction	0.34
2	153	80	305	124	105	158		
LEA								
0	117	87	162	121	98	157	Time	0.0003
0.5	146	124	173	147	129	202	Time+TNFα	0.26
1	203	193	211	154	20	187	Interaction	0.10
2	163	104	195	165	142	195		
PGE_2_EA^†^								
0	977	594	1736	967	434	2274	Time	0.0002
0.5	636	452	1008	803	379	1712	Time+TNFα	0.98
1	545	422	883	488	304	850	Interaction	0.76
2	474	250	801	482	392	562		
PGD_2_								
0	366	329	398	417	374	455	Time	0.0010
0.5	383	344	434	390	347	429	Time+TNFα	0.17
1	372	295	415	410*	373	446	Interaction	0.0095
2	353	312	413	317	285	381		
PGE_2_								
0	241	193	282	253	211	318	Time	0.047
0.5	236	223	251	218	144	261	Time+TNFα	0.99
1	212	178	253	241	217	275	Interaction	0.061
2	224	182	250	201	137	237		
TXB_2_^†^								
0	4398	2547	5425	3003	2759	3503	Time	<0.0001
0.5	4202	2765	6731	5087	3967	6204	Time+TNFα	0.59
1	2841	2505	3083	3085	2866	3360	Interaction	0.0048
2	2632	2027	3050	2277	1777	2614		
15-HETE								
0	15	0	47	25	0	152	Time	<0.0001
0.5	242	179	287	139	80	191	Time+TNFα	0.017
1	211	144	317	135	35	188	Interaction	0.011
2	104	13	172	86	18	152		

Values are means with ranges of the concentrations for aliquots taken at the time points shown. A linear mixed model (function lme in the package nlme for R) was used as described by Field et al. [41], where the factors are added sequentially. The P values are for the ANOVA of the baseline, Time, Time+TNFα and interaction models. In some cases (marked with ^†^), the residual plots were not acceptable, but this was rectified by using log-transformed data instead. At a 5% false discovery rate [42], the critical P value was 0.021. Sample sizes were n = 6 for both TNFα and controls.

*One extreme value (3843 pM) was excluded. Note that in this experiment, 2-AG was not detected in the medium.

The methodology used [37] also measures 2-AG levels in biological samples. In the first experiment, 2-AG was quantitated in both medium and cell lysates, and there was no significant effect of TNFα (Fig 4A, Table 3). In the second experiment, no 2-AG peak could robustly be quantitated. In the striatum, elevated AEA levels reduce endogenous 2-AG by a transient receptor potential vanilloid 1 (TRPV1) mediated mechanism [47]. DU145 cells express TRPV1 [48] and it is thus reasonable to conclude that the loss of 2-AG signal in our experiment is due to the added exogenous AEA.

## Discussion

The aim of the study was to assess the effect of TNFα treatment upon AEA turnover in DU145 cells, since little is known about the regulatory effects of TNFα upon the eCB system, in contrast to the large body of data concerning effects of eCBs, synthetic, and plant-derived cannabinoids upon the TNFα and other cytokines [25,26] (review see [49]). There were two main findings that are discussed below:

*TNFα treatment affects the balance of AEA catabolic enzymes in DU145 cells*. Consistent with the literature [30], TNFα treatment of the cells increased the expression of *PTGS2*. Levels of *PTGS2* expression were low, and we could not detect COX-2 in Western blot experiments. However, Subbarayan et al. [30] were able to detect the protein in both Western blot and immunofluorescence experiments. They found increased expression of COX-2 as early as 0.5 h after TNFα treatment in the cells, and also reported an increased expression of COX-2 in androgen-insensitive PC3 cells, but not in the androgen-sensitive LNCaP cells. In contrast to the increase in PTGS2 expression, we found that *FAAH* and *NAAA* expression at the mRNA level were not affected by the TNFα treatment. The functional assay of [Et-^3^H]AEA hydrolysis also failed to show significant changes following TNFα treatment. Importantly, the [Et-^3^H]AEA hydrolysis was completely inhibited by the selective FAAH inhibitor URB597, confirming that FAAH was the primary enzyme responsible for the hydrolysis of externally administered AEA in these cells.

In theory, the net result of the increased COX-2 expression relative to the FAAH (and NAAA) expression following TNFα treatment should be a greater production of PG-EAs. However, the low levels of COX-2 in the cells meant that PG-EAs (and PGs themselves) were only detected in the lipidomics experiments when the cells had been incubated for 30 min with 100 nM AEA. In their experiment, Subbarayan et al. [30] detected PGE_2_ using an enzyme immunoassay kit and reported a 1.8-fold increase in levels following TNFα treatment. It is possible that the basal expression of COX-2 was higher in their DU145 cells than in our cells. These authors did not look for PG-EAs (although they may inadvertently have measured it together with PGE_2_ in their immunoassay [36]). We identified the PG-EA that was detected in our experiments as PGE_2_-EA rather than PGF_2α_-EA. This is an interesting finding given that PGE_2_-EA reduces LPS-induced TNFα production by monocytes [50] whereas PGF_2α_-EA is pro-inflammatory in nature [51]. It sounds counter-intuitive from the tumour cells point of view that they should produce anti- rather than pro-inflammatory compounds, but the levels were low and the reduction in NAPE-PLD expression, by reduction of the synthesis of the AEA precursor, would work against a PGE_2_-EA → TNFα feedback loop in the cells.

Although not part of the main aim of the study, we also investigated the effect of TNFα treatment on the other endocannabinoid, 2-AG. At the level of mRNA, TNFα reduced the levels of the synthetic enzymes DAGL-α and –β, but this was not sufficient to affect the observed levels of 2-AG in either the medium or the cell lysates. More pronounced pharmacological inhibition of 2-AG synthesis, however, does reduce 2-AG levels in these cells, and this is accompanied by an increased invasivity of the cells across matrigel-coated transwells [14].

*COX-2 does not gate the uptake of AEA in LPS+IFNγ-treated RAW264*.*7 cells*. The manner in which AEA is accumulated in cells has been a matter of considerable debate, in particular with respect to whether or not there is a plasma membrane bidirectional transporter protein [52,53]. It is clear, however, that in cells expressing FAAH, this enzyme gates the accumulation by reducing the intracellular free AEA concentration and hence preserving the gradient across the plasma membrane [8]. Very little work has been undertaken on the uptake of AEA into prostate cancer cells, but PC3 cells, which express low levels of FAAH [54], unsurprisingly do not shown this phenomenon, with uptake in the absence and presence of FAAH inhibitors being very similar [53]. In contrast, rat Dunning AT1 prostate cancer cells express FAAH, and AEA uptake into these cells is reduced upon FAAH inhibition [55]. The DU145 cells resemble more the AT1 cells than the PC3 cells in this respect.

Given that FAAH, by removing intracellular AEA, can maintain AEA uptake, a similar property might be expected for other enzymes using AEA as a substrate, such as COX-2, since COX-2-catalysed cyclooxygenation of AEA would deplete local AEA levels. This phenomenon should be expected to be extra prominent in cases of increased COX2 expression. The COX-2 levels were too low in the DU145 cells to test this hypothesis, but in the LPS+IFNγ-treated RAW264.7 cells, which were included in the study as a positive control for COX-2 (albeit with the limitation that they are from mouse, rather than man), the expression levels are very high. As a rough guide, the k_cat_ and K_m_ values for human COX-2 towards AEA are 4 s^-1^ and 62 μM respectively, at pH 8 [56], whereas the corresponding values for rat FAAH towards this substrate are 4.8 s^-1^ and 17 μM, respectively [57]. The very much higher expression of COX-2 than FAAH in the LPS+IFNγ-treated RAW264.7 cells, at least at the mRNA level, would suggest that COX-2 will play a significant role in AEA metabolism. Indeed, we were able to detect a peak eluting in the same region as the PG-EA analogue bimatoprost following incubation of the LPS+IFNγ-treated (but not control) RAW264.7 cells with [Ara-^3^H]AEA. If COX-2 gated AEA uptake in these cells in the same way as FAAH, then its induction by LPS+IFNγ treatment should increase the rate of uptake, while pharmacological inhibition of the enzyme by flurbiprofen should reduce the uptake back to the level seen for the control RAW264.7 cells. Neither event occurred, in contrast to the clear effect of the FAAH inhibitor URB597 in the cells. It is at first sight surprising that one catabolic enzyme can gate AEA uptake in this way whereas another one cannot. However, for 2-AG, a different pattern is seen, where the hydrolytic enzymes do not regulate the uptake, whereas down-stream processes (incorporation of the arachidonic acid into the phospholipids) are involved [58,59]. Thus, the intracellular processes regulating AEA and 2-AG uptake are complex and different for the two endocannabinoids despite the fact that they are both arachidonoyl derivatives.

In conclusion, the present study has provided novel data on the effects of TNFα treatment on AEA catabolic pathways in DU145 prostate cancer cells and suggest that the balance of catabolism is tilted towards COX-2 by this treatment, although the low expression of this enzyme in the cells limits the functional consequence of such a tilt. Further studies in other prostate cancer cells, ideally with different basal levels of the synthetic and degradative enzymes and with other inflammatory mediators, are warranted in order to shed more light upon the relationship between inflammation and the disturbed eCB system in prostate cancer and the functional consequence of increased COX-2 levels upon the levels and metabolism of AEA.
